# Relationship between functional status and fatigue after COVID-19 infection: a multicenter study from Türkiye

**DOI:** 10.55730/1300-0144.5831

**Published:** 2024-07-14

**Authors:** İpek CANDEMİR, Pınar ERGÜN, Dicle KAYMAZ, Mustafa Engin ŞAHİN, İpek ÖZMEN, Elif YILDIRIM, Aslı GÖREK DİLEKTAŞLI, Büşra YİĞİTLİLER, Ayten ODABAŞ, Deniz KIZILIRMAK, Seçil SARI, Celalettin KORKMAZ, Cantürk TAŞÇI, Yakup ARSLAN, Sema SAVCI, Buse KAHRAMAN, Aylin TANRIVERDİ, Can SEVİNÇ, Melda SAĞLAM, Deniz İNAL İNCE, Naciye VARDAR YAĞLI, Ebru KÜTÜKÇÜ, Dilber DURMAZ, Neslihan DURUTÜRK, Gaye ULUBAY, Lütfiye KILIÇ, Benan ÇAĞLAYAN, Aylin MORAY, Sabri Serhan OLCAY, Güven ÖZKAYA

**Affiliations:** 1 Department of Chest Diseases, Ankara Atatürk Sanatoryum Education and Research Hospital, University of Health Sciences, Ankara, Turkiye; 2 Department of Chest Diseases, Süreyyapaşa Chest Diseases and Surgery Education and Research Hospital, İstanbul Provincial Directorate of Health, İstanbul, Turkiye; 3 Department of Chest Diseases, Faculty of Medicine, Uludağ University, Bursa, Turkiye; 4 Department of Chest Diseases, Faculty of Medicine, Celal Bayar University, Manisa, Turkiye; 5 Department of Chest Diseases, Faculty of Medicine, Necmettin Erbakan University, Konya, Turkiye; 6 Department of Chest Diseases, Faculty of Medicine, Gülhane University, Ankara, Turkiye; 7 Department of Physical Therapy and Rehabilitation, Faculty of Physical Therapy and Rehabilitation, Dokuz Eylül University, İzmir, Turkiye; 8 Department of Chest Diseases, Faculty of Medicine, Dokuz Eylül University, İzmir, Turkiye; 9 Department of Physical Therapy and Rehabilitation, Faculty of Physical Therapy and Rehabilitation, Hacettepe University, Ankara, Turkiye; 10 Department of Chest Diseases, Bandırma Public Hospital, Balıkesir, Turkiye; 11 Department of Physical Therapy and Rehabilitation, Institute of Health Sciences, Başkent University, Ankara, Turkiye; 12 Department of Chest Diseases, Faculty of Medicine, Başkent University, Ankara, Turkiye; 13 Department of Chest Diseases, Faculty of Medicine, Koç University, İstanbul, Turkiye; 14 Department of Chest Diseases, Çorum Public Hospital, Çorum, Turkiye; 15 Department of Chest Diseases, Faculty of Medicine, Muğla University, Muğla, Turkiye; 16 Department of Statistics, Faculty of Medicine, Uludağ University, Bursa, Turkiye

**Keywords:** Dyspnea, functional capacity, quality of life

## Abstract

**Background/aim:**

Symptoms of COVID-19 may persist for months. One of the persistent symptoms of COVID-19 is fatigue, which reduces functional status. The relationship between fatigue, functional status, and various other factors has received little attention, which this study aims to address..

**Materials and methods:**

Patients with COVID-19 infection were included in this multicenter cross-sectional study. Age, sex, body mass index (BMI), marital status, smoking status, presence and duration of chronic disease, comorbidity index, regular exercise habits, time since COVID-19 diagnosis, hospitalization status, length of hospital stay, intubation status, home oxygen therapy after discharge, participation in a pulmonary rehabilitation program, presence of dyspnea, presence of cough, presence of sputum, and modified Medical Research Council, Post-COVID Functional Status (PCFS), Fatigue Severity Scale (FSS), and EQ-5D-5L Questionnaire scores were recorded.

**Results:**

We enrolled 1095 patients, including 603 (55%) men and 492 (45%) women with a mean age of 50 ± 14 years. The most common chronic lung disease was COPD (11%) and 266 (29%) patients had nonpulmonary disease. The median time elapsed since COVID-19 diagnosis was 5 months; the hospitalization rate was 47%. The median PCFS grade was 1 (0–4) and the median FSS score was 4.4 (1–7). The PCFS and FSS were positively correlated (r = 0.49, p < 0.01; OR: 1.88, 95% CI: 1.68–2.10). Both functional status and fatigue were associated with quality of life, which was lower in older patients, those with higher BMI, those with systemic disease, those not exercising regularly, and those with more severe COVID-19 infection (defined by dyspnea, pneumonia as indicated by computed tomography, hospitalization, length of stay, ICU admission, intubation, and the need for home oxygen after discharge).

**Conclusion:**

Fatigue may cause poorer functional status regardless of the time since COVID-19 diagnosis. In this study, patients with FSS scores of >4.78 showed moderate to severe functional limitations. It is important to address modifiable patient risk factors and reduce the severity of COVID-19 infection.

## 1. Introduction

Coronavirus disease 2019 (COVID-19) emerged in China in December 2019 and was later declared a pandemic. As of 2022, more than 500 million people had been infected with COVID-19 and over 6 million had died.^1^ Symptoms of COVID-19 may persist for weeks or months. Although many patients regain their preinfection health status, 10% have ongoing symptoms including dyspnea, exercise intolerance, fatigue, depression, anxiety, and cardiac, pulmonary, and renal anomalies [1]. Over 30% of all patients (including those who are asymptomatic) and about 80% of those who are hospitalized experience post-COVID symptoms [2,3]. The natural course of COVID-19 infection, prognosis, and long-term consequences including poor health and reduced health-related quality of life have not been elucidated in detail [1,4]. Typically, multisystemic symptoms reduce functional performance and compromise the performance of activities of daily living [5]. Symptom severity varies widely. The Post-COVID-19 Functional Status (PCFS) scale was established by Klok et al. [6] for use at the time of hospital discharge or thereafter [4]. One of the most important and persistent symptoms of COVID-19 is fatigue, which reduces functional status through its effects on body composition and physical fitness. The impact of fatigue also differs by sex and comorbidities [7,8]. The relationships of fatigue with functional status and various other factors has received little attention, which we aim to address in this study.

## 2. Materials and methods

### 2.1. Study population

**Figure 1:**
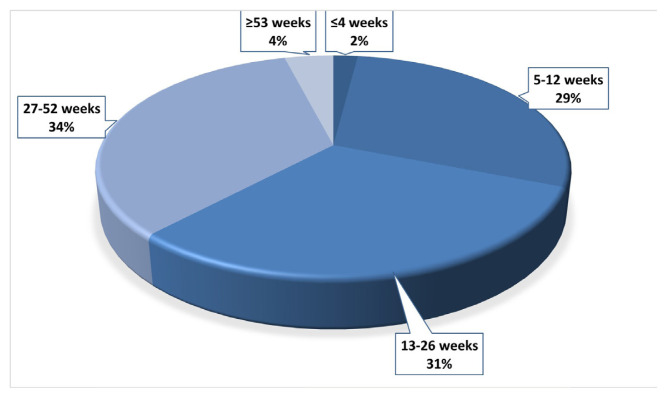
Percentage of patients according to time since COVID-19 diagnosis.

Patients with a history of COVID-19 infection referred to us by 13 outpatient clinics and pulmonary rehabilitation (PR) centers or units were enrolled in this study between 1 April 2021 and 31 December 2021 as shown in Figure 1.

Inclusion criteria included a history of COVID-19 infection confirmed by polymerase chain reaction (PCR), medical examination, or thoracic computed tomography (CT) and no acute infection at the time of evaluation according to medical history, serum C-reactive protein level, X-ray, or CT.

Exclusion criteria included refusal to participate/sign the informed consent form, acute myocardial infarction, unstable angina, uncontrolled arrhythmia, hemodynamic instability, syncope, active endocarditis, acute myocarditis or pericarditis, symptomatic and severe aortic stenosis, uncontrolled heart failure, acute pulmonary embolism or pulmonary infarction, lower-extremity thrombosis, dissected aneurysm, any mental disorder, neurological or rheumatological disorders, acute exacerbation of a respiratory disease such as chronic obstructive pulmonary disease (COPD), bronchiectasis, asthma, or an inability to complete the questionnaires.

### 2.2. Study design

This multicenter cross-sectional study involved 13 units in nine university, public, and research hospitals. Written informed consent was obtained from patients and ethical approval (Gülhane Education and Research Hospital Ethic Committee, number: 6135, 2021-242) was granted by the Ministry of Health on 26 February 2021.

#### 2.2.1. Outcome Measures

For primary outcomes, the validated PCFS scale was used to assess functional status/limitations (grade 0: “No functional limitations”; grade 4: “Severe functional limitations”; grade 5: “Death”) [9,10]. Fatigue was evaluated using the validated self-report Fatigue Severity Scale (FSS), which includes nine items, each scored from 1 to 7 (1: “completely disagree”; 7: “completely agree”) [10,11].

For secondary outcomes, age, sex, height, body weight, marital status, employment status, smoking status and amount, chronic disease status and duration, regular exercise habits, time since COVID-19 diagnosis, COVID-19 pneumonia status (as revealed by thoracic CT), hospitalization, length of hospital and/or intensive care unit (ICU) stays, intubation status, home oxygen therapy status, participation in a PR program, dyspnea, cough, sputum production, and quality of life scores were recorded. All measurements were performed by the research coordinator in a well-ventilated room. The coordinator wore protective personal equipment and maintained social distancing. Hospital data were used to determine length of stay.

The EQ-5D-5L quality-of-life scale consists of a descriptive section and questions answered using a visual analog scale (VAS). The first section explores mobility, self-care, engagement in usual activities, pain/discomfort, and anxiety/depression experiences during the current day. The answer options are “No problem,” “Mild problem,” “Moderate problem,” “Severe problem,” and “Extreme problem.” In the second part, with six questions, participants rate their current health status from 0 and 100, with higher scores indicating better perceived health. The Turkish version of the scale, translated by the EuroQol group, were used in some studies.^2^

Comorbidities were scored using the Charlson Comorbidity Index (CCI). Scores increase by 1 point every 10 years after the age of 50 years. The following conditions were assessed (with CCI points given in parentheses): history of definite or probable myocardial infarction (1); congestive heart failure (1); peripheral vascular disease (1); cerebrovascular disease (1); dementia (1), COPD (1); connective tissue disease (1); peptic ulcer (1); liver disease (mild, 1; moderate to severe, 3); diabetes mellitus (1); hemiplegia (2); moderate-to-severe chronic kidney disease (2); solid tumor (localized, 2; metastasis 6); leukemia (2); malignant lymphoma (2); and acquired immune deficiency syndrome (6) [12]. Body mass index (BMI) was recorded as weight (kg) divided by height squared (m^2^). Dyspnea was assessed using the modified Medical Research Council (mMRC) scale [13]. Regular exercise was defined as 150–300 min of moderate-intensity activity, such as walking briskly, each week.^3^

### 2.3. Statistical analyses

We used the Shapiro–Wilk test to determine whether the data were normally distributed. The results are presented as mean ± standard deviation, median (range), or frequency (percentage). Normally distributed data were compared using the independent samples t-test or one-way ANOVA. The Kruskal–Wallis and Mann–Whitney U tests were used to compare data that were not normally distributed. Bonferroni multiple-comparisons testing was used as appropriate. Categorical variables were compared between groups using the Pearson chi-square test and Fisher exact test. Correlations between variables were analyzed by Spearman correlation. We performed multiple linear and ordinal regression analyses. Values of p < 0.05 were considered to reflect significance. All analyses were performed with IBM SPSS Statistics 28 (IBM Corp., Armonk, NY, USA).

## 3. Results

We enrolled 1095 patients, including 603 (55%) men and 492 (45%) women with a mean age of 50 ± 14 years. The most common chronic lung disease was COPD (11%), and 266 (29%) patients had nonpulmonary systemic disease. The median time elapsed since COVID-19 diagnosis was 5 months; the hospitalization rate was 47% (Tables 1 and 2). The number of patients with a history of COVID-19 infection within the last 4 weeks was 180 (2%), while 269 (29%) had a history of COVID-19 within the last 5–12 weeks, 286 (31%) within the last 13–26 weeks, 321 (34%) within the last 27–52 weeks, and 39 (4%) within the last ≥53 weeks. The percentages of patients according to time since COVID-19 diagnosis are shown in Figure 1.

### 3.1. Relationship between PCFS grade and FSS score

**Figure 2:**
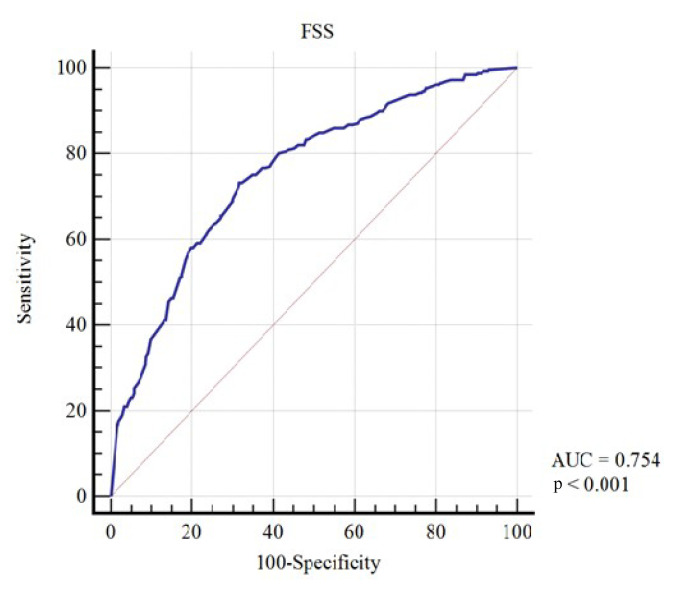
Receiver operating characteristic curve for prediction of FSS based on PCFS grade of 3 or 4.

The median PCFS grade was 1 (range: 0–4) and the median FSS score was 4.4 (range: 1–7). The PCFS and FSS were positively correlated (r = 0.49, p < 0.01; odds ratio [OR]: 1.88, 95% confidence interval [CI]: 1.68–2.10). The receiver operating characteristic (ROC) curve for prediction of the FSS score based on PCFS grade of 3 or 4 is shown in Figure 2. In patients with PCFS grades of 0–2, the mean FSS score was 3.9 ± 1.6. In patients with PCFS grades of 3 or 4, the mean FSS score was 5.3 ± 1.3 (FSS cutoff score: 4.78; 73.1% sensitivity).

### 3.2. Relationships between PCFS grade and other parameters

PCFS grades were significantly higher among patients who were married, had systemic or respiratory disease, had positive CT findings, or had been hospitalized, intubated, treated in the ICU, or required home oxygen (p < 0.01 for all). They were also higher among those with dyspnea (p < 0.01), cough (p = 0.006), and sputum (p = 0.048) and those who did not exercise regularly before COVID-19 infection (p = 0.001). PCFS grades were significantly reduced in active smokers compared to former smokers and never-smokers (p < 0.05). PCFS grades did not vary by sex (p = 0.20), employment status (p = 0.15), or PR status (p = 0.78). We found significant correlations between PCFS grade and age (r = 0.354, p < 0.01), BMI (r = 0.146, p < 0.01), mMRC scale score (r = 0.631, p < 0.01), length of hospital stay (r = 0.386, p < 0.01), length of ICU stay (r = 0.212, p < 0.01), CCI score (r = 0.192, p < 0.01), scores on all domains of the EQ-5D-5L (p < 0.01), and EQ-5D-5L VAS scores (r = −0.525, p < 0.01). PCFS grade did not correlate with the duration of respiratory disease (p = 0.11), amount of smoking (r = 0.025, p = 0.45), or time since COVID-19 diagnosis (p = 0.78). Ordinal regression analyses showed that current smoking, COVID-19 pneumonia evident on CT scans, mMRC score, and EQ-5D-5L scores for the domains of mobility, self-care, performance of usual activities, and anxiety/depression and EQ-5D-5L VAS scores were associated with PCFS grade. These results are shown in Table 3.

### 3.3. Relationships between FSS score and other parameters

The FSS score was significantly elevated in women (p < 0.01), patients who were married (p = 0.001), unemployed patients (p < 0.01), patients with respiratory or systemic disease (p = 0.001), patients with positive CT findings (p < 0.01), patients who were hospitalized (p < 0.01) or admitted to an ICU (p < 0.01), patients who required home oxygen (p < 0.01), patients with dyspnea (p < 0.01), patients with cough (p = 0.005), patients with sputum (p = 0.017), and patients who did not exercise regularly before COVID-19 infection (p < 0.01). FSS scores did not differ by smoking status (p = 0.52), intubation status (p = 0.34), or PR status (p = 0.68). FSS scores correlated with age (r = 0.135, p < 0.01), BMI (r = 0.092, p = 0.002), mMRC scale score (r = 0.357, p < 0.01), duration of respiratory disease (r = 0.092, p = 0.007), length of hospital stay (r = 0.185, p < 0.01), length of ICU stay (r = 0.105, p = 0.001), CCI score (r = 0.193, p < 0.01), scores on all EQ-5D-5L domains (p < 0.01 for all domains, r = 0.496 for mobility / r = 0.441 for self-care / r = 0.505 for usual activities / r = 0.380 for pain-discomfort / r = 0.424 for anxiety-depression) and EQ-5D-5L VAS scores (r = −0.471, p < 0.01), but not with amount of smoking (p = 0.50) or time since COVID-19 diagnosis (p = 0.25). Linear regression analyses showed that female sex, COPD, EQ-5D-5L domain scores (mobility, usual activities, and anxiety/depression), and EQ-5D-5L VAS scores were associated with FSS scores. These results are shown in Table 4.

## 4. Discussion

We found that even moderate functional status was associated with fatigue regardless of the time since COVID-19 diagnosis. A one-point increase in FSS score increased the likelihood of a one-grade increase on the PCFS scale by 1.882-fold. The cutoff FSS score for patients with moderate-to-severe functional capacity limitations was 4.78 and the mean FSS score was 5.3 ± 1.3. Both functional status and fatigue were associated with quality of life, which was lower in older patients, those with higher BMI, those with respiratory or systemic disease, those with comorbidities, those not exercising regularly, and those with more severe COVID-19 infection (defined by dyspnea, pneumonia as indicated by CT, hospitalization, length of stay, ICU stay, intubation, and the need for home oxygen after discharge).

After COVID-19 infection, functional status may deteriorate. The PCFS scale measures all functional limitations. It should be administered at discharge, at 4 and 8 weeks to monitor recovery, and at 6 months to evaluate functional sequelae [6]. More of our patients had negligible (grade 1) than mild (grade 2) limitations, perhaps reflecting the long intervals since COVID-19 diagnosis (>4 weeks and >6 months for 98% and 36% of patients, respectively). In our study, the examination of patient groups using the PCFS scale revealed substantial associations with demographic, clinical, and lifestyle factors. Higher PCFS grades, indicative of a more compromised functional status, were notably linked to marital status, underlying systemic or respiratory diseases, positive CT findings, and severe clinical manifestations necessitating hospitalization, intubation, or ICU admission. Additionally, the presence of symptoms such as dyspnea, cough, and sputum coupled with a lack of regular exercise before COVID-19 infection correlated with elevated PCFS grades. Similarly, the analysis of patient groups based on FSS scores revealed significant associations with various demographic and clinical factors, providing valuable insights into post-COVID-19 fatigue levels. Elevated FSS scores, indicative of heightened fatigue, were notably associated with female sex, marital status, unemployment, and the presence of respiratory or systemic diseases. Furthermore, positive CT findings, hospitalization, admission to the ICU, the need for home oxygen, and the existence of symptoms such as dyspnea, cough, or sputum were all linked to increased FSS scores. Importantly, FSS scores exhibited no significant differences based on smoking status, intubation status, or participation in PR programs. These findings collectively contribute to our understanding of the diverse determinants influencing post-COVID-19 fatigue and functional outcomes. Additionally, the findings underscore the importance of considering diverse factors in assessing and managing the long-term functional status of individuals recovering from COVID-19.

Limited functional capacity can be caused by persistent symptoms, the most common of which is fatigue (50%–65%) [5]. The association between functional status and fatigue is multifactorial; body composition, physical fitness, comorbidities, and sex are all important [8,9]. Fatigue and functional impairment are highly associated; both are influenced by pain and by demographic and clinical factors in patients with cancer. Although it has proven difficult to distinguish fatigue from factors such as depression and sleep disorders and to assess how comorbidities contribute to fatigue, post-COVID-19 fatigue is defined as a decrease in physical and/or mental performance caused by changes in central, psychological, or “peripheral” factors [14]. The relationship between fatigue (and related factors) and functional status in COVID-19 survivors has not been properly explored.

COVID-19 survivors with moderate-to-severe limitations in functional capacity exhibited moderate-to-severe fatigue in a study on stroke patients in whom fatigue was categorized as absent (FSS score of <4), moderate (4–4.9), or severe (≥5) [15]. We found that poor quality of life was the strongest risk factor for limited functional status and fatigue. Poor functional status and fatigue were associated with poor mobility, an inability to perform usual activities, and anxiety/depression. One study found that the PCFS grade was related to fatigue, quality of life, and functional performance [16]. In another study, the score for the “usual activities” domain of the EQ-5D-5L questionnaire was the domain score most strongly associated with PCFS grade [10]. We found that mobility was most strongly associated with FSS score and exhibited the second-strongest association after smoking with PCFS grade. Smoking causes inflammation and respiratory and systemic diseases, complicating disease management. One study found that subjects with PCFS grade of 0 were older than those with higher grades. Additionally, the grade 4 group had lower BMI values than the other groups [10].

In a previous study, decreased functional status was associated with living alone and comorbidities [10]. Risk factors for fatigue were older age, female sex, and more symptoms [17]. We found that older age, being married, higher BMI, and respiratory and systemic diseases were associated with poorer functional status and more fatigue. The two primary characteristics that increased the risk factors for fatigue were COPD and female sex. Being married was probably a risk factor because married people tended to be older. No regular exercise before COVID-19 infection increased fatigue and reduced functional status; a healthy lifestyle and regular exercise are important. Another study found that obesity, older age, and comorbidities were risk factors for severe COVID-19 disease [18], as indicated by hospitalization [19], ICU admission [20], the duration of hospital stay, viral shedding [21], and dyspnea during hospitalization [19,21]. All of these factors were associated with fatigue during follow-up, but the length of hospital stay was the only predictor of poorer functional status at the time of hospital discharge [16]. We found that dyspnea, COVID-19 pneumonia as indicated by CT, hospitalization, length of hospital stay, ICU admission, intubation, and the need for home oxygen after discharge were related to poor functional status and fatigue, whereas PR was not; this may have been because PR was rarely performed.

The limitations of this study included patient heterogeneity and a lack of vaccination data. Additionally, we did not perform functional capacity or exercise tests. The multicenter design and large number of patients were strengths of the study. As an additional limitation, it is important to acknowledge the possibility of selection bias within the patient population. The individuals under study were not summoned for routine check-ups; rather, they sought medical attention at the hospital due to the presence of comorbidities and specific complaints. Since there is a lack of information pertaining to the preinfection PCFS/FSS scores of these patients prior to their COVID-19 diagnoses, asserting a causal relationship would be inherently inaccurate. Consequently, the observed fatigue and diminished functional status in this patient cohort may be attributed to their existing comorbidities, potentially unrelated to COVID-19. Additionally, it is plausible that individuals experiencing such symptoms after COVID-19 may have been admitted to other medical departments such as family medicine or internal medicine.

Fatigue may cause poorer functional status regardless of the time since COVID-19 diagnosis. In this study, patients with FSS scores of >4.78 showed moderate-to-severe functional limitations. It is important to address modifiable patient risk factors and reduce the severity of COVID-19 infection. Weight control, regular exercise, smoking cessation, management of anxiety and depression, treatment of comorbidities, and vaccination are required. Given its multidisciplinary and comprehensive nature, PR is useful for patients with chronic respiratory disease.

## Figures and Tables

**Table 1:** Demographic features and chronic disorders.

Age, years	50 ± 14
Sex, M/F, n (%)	603 (55%)/492 (45%)
BMI, kg/m^2^	28.1 ± 5.1
Marital status, n (%)	Single: 162 (14.8%); Married: 933 (74.2%)
Employment status, n (%)	Unemployed: 294 (27%); Employed: 532 (49%); Retired: 269 (24%)
Smoking status, n (%)	Never: 616 (56%); Active: 161 (15%); Former: 318 (29%)
Amount of smoking, pack years	4 (1–120)
Chronic respiratory diseases, n (%)	None: 853 (77.9%); COPD: 125 (11.4 %); Asthma: 89 (8.1%); ILD: 8 (0.7%); Sleep disorders: 5 (0.5%); Bronchiectasis: 9 (0.8%); Lung cancer: 5 (0.5%); Others: 1 (0.1%)
Duration of respiratory disease, years	7 (0.5–30)
Presence of nonrespiratory systemic disease, n (%)	266 (29%)
CCI score	0 (0–6)

BMI: Body mass index; COPD: chronic obstructive pulmonary disease; ILD: interstitial lung disease; CCI: Charlson Comorbidity Index.

**Table 3:** Parameters associated with PCFS grade according to regression analyses.

Parameters	p	OR	95% CI
Current smoking	0.034	2.767	1.080–7.088
CT findings	0.001	0.443	0.277–0.706
mMRC score of 0	0.005	0.097	0.019–0.486
mMRC score of 1	<0.01	0.078	0.031–0.199
mMRC score of 2	<0.01	0.222	0.095–0.516
mMRC score of 3	<0.01	0.353	0.153–0.816
EQ-5D-5L Mobility	<0.01	1.999	1.403–2.850
EQ-5D-5L Self-care	0.021	1.539	1.066–2.222
EQ-5D-5L Usual activities	0.005	1.750	1.188–2.577
EQ-5D-5L Anxiety/Depression	<0.01	1.708	1.284–2.272
VAS	0.026	0.982	0.966–0.998

PCFS: Post-COVID Functional Status; CT: computed tomography; mMRC: modified Medical Research Council; VAS: visual analog scale.

**Table 4:** Parameters associated with FSS score according to regression analyses.

Parameters	Standardized beta coefficient	p
Sex: male	−0.121	<0.01
Chronic respiratory disease: COPD	0.064	0.029
EQ-5D-5L Mobility	0.156	0.001
EQ-5D-5L Usual activities	0.114	0.019
EQ-5D-5L Anxiety/Depression	0.095	0.009
VAS	−0.223	<0.01

FSS: Fatigue Severity Score; COPD: chronic obstructive pulmonary disease; VAS: visual analog scale.

## Footnotes

1 World Health Organization (2022). Coronavirus (COVID-19) [online]. Website https://covid19.who.int [accessed 08 August 2022].

2 EuroQol Group (2015). EQ-5D-5L User Guide. Basic Information on How to Use the EQ-5D-5L Instrument, Version 2.1 [online]. Website https://euroqol.org/register/obtain-eq-5d/available-versions/ [accessed: 11 August 2022].

3 US Department of Health and Human Services (2018). Physical Activity Guidelines for Americans, 2nd Edition [online]. Website https://health.gov/sites/default/files/2019-09/Physical_Activity_Guidelines_2nd_edition.pdf [accessed: 08 August 2022].

## References

[1] Nalbandian A Sehgal K Gupta A Madhavan MV McGroder C Post-acute COVID-19 syndrome Nature Medicine 2021 27 601 615 10.1038/s41591-021-01283-z PMC8893149 33753937

[2] Tenforde MW Kim SS Lindsell CJ Rose EB Shapiro NI Symptom duration and risk factors for delayed return to usual health among outpatients with COVID-19 in a multistate health care systems network - United States, March–June 2020 MMWR Morbidity and Mortality Weekly Report 2020 69 30 993 998 10.15585/mmwr.mm6930e1 32730238 PMC7392393

[3] Huang Y Pinto MD Borelli JL Mehrabadi MA Abrihim H COVID symptoms, symptom clusters, and predictors for becoming a long-hauler: looking for clarity in the haze of the pandemic Clinical Nursing Research 2022 31 8 1390 1398 10.1177/10547738221125632 36154716 PMC9510954

[4] Carfì A Bernabei R Landi F Gemelli Against COVID-19 Post-Acute Care Study Group Persistent symptoms in patients after acute COVID-19 JAMA 2020 324 6 603 605 10.1001/jama.2020.12603 32644129 PMC7349096

[5] Antoniou KM Vasarmidi E Russell AM Andrejak C Crestani B European Respiratory Society statement on long COVID follow-up European Respiratory Journal 2022 60 2102174 10.1183/13993003.02174-2021 35144991 PMC9349784

[6] Klok FA Boon GJAM Barco S Endres M Geelhoed JJM The Post-COVID-19 Functional Status (PCFS) Scale: a tool to measure functional status over time after COVID-19 European Respiratory Journal 2020 56 1 2001494 10.1183/13993003.01494-2020 32398306 PMC7236834

[7] Egerton T Chastin SF Stensvold D Helbostad JL Fatigue may contribute to reduced physical activity among older people: an observational study Journals of Gerontology Series A, Biological Sciences and Medical Sciences 2016 71 670 676 10.1093/gerona/glv150 26347508

[8] Aldhahi MI Alshehri MM Alqahtani F Alqahtani AS A pilot study of the moderating effect of gender on the physical activity and fatigue severity among recovered COVID-19 patients PLoS One 2022 17 7 e0269954 10.1371/journal.pone.0269954 35830386 PMC9278785

[9] Kütükçü EC Çakmak A Kınacı E Uyaroğlu OA Yağlı NV Reliability and validity of the Turkish version of Post-COVID-19 Functional Status Scale Turkish Journal of Medical Sciences 2021 51 5 2304 2310 10.3906/sag-2105-125 34392673 PMC8742502

[10] Machado FV Meys R Delbressine JM Vaes WA Goërtz YMJ Construct validity of the Post-COVID-19 Functional Status Scale in adult subjects with COVID-19 Health and Quality of Life Outcomes 2021 19 1 40 10.1186/s12955-021-01691-2 33536042 PMC7856622

[11] Gencay-Can A Can SS Validation of the Turkish version of the fatigue severity scale in patients with fibromyalgia Rheumatology International 2012 32 1 27 31 10.1007/s00296-010-1558-3 20658235

[12] Charlson ME Pompei P Ales KL MacKenzie CR A new method of classifying prognostic comorbidity in longitudinal studies: development and validation Journal of Chronic Diseases 1987 40 373 383 10.1016/0021-9681(87)90171-8 3558716

[13] Wu Q Hou X Li H Guo J Li Y A follow-up study of respiratory and physical function after discharge in patients with redetectable positive SARS-CoV-2 nucleic acid results following recovery from COVID-19 International Journal of Infectious Diseases 2021 107 5 11 10.1016/j.ijid.2021.04.020 33857606 PMC8056475

[14] Rudroff T Fietsam AC Deters JR Bryant AD Kamholz J Post-COVID-19 fatigue: potential contributing factors Brain Sciences 2020 10 12 1012 10.3390/brainsci10121012 33352638 PMC7766297

[15] Lerdal A Bakken LN Rasmussen EF Beiermann C Ryen S Physical impairment, depressive symptoms and pre-stroke fatigue are related to fatigue in the acute phase after stroke Disability and Rehabilitation 2010 33 4 334 342 10.3109/09638288.2010.490867 20521900

[16] Leite CT Carvalho L Queiroz M Farias MSQ Cavalheri V Can the post-COVID-19 functional status scale discriminate between patients with different levels of fatigue, quality of life and functional performance? Pulmonology 2022 28 220 223 10.1016/j.pulmoe.2022.01.001 35120866 PMC8752288

[17] Townsend L Dowds J O’Brien K Sheill G Dyer AH Persistent poor health post-COVID-19 is not associated with respiratory complications or initial disease severity Annals of the American Thoracic Society 2021 18 6 997 1003 10.1513/AnnalsATS.202009-1175OC 33413026 PMC8456724

[18] Alberca RW Oliveira LM Branco ACCC Pereira NZ Sato MN Obesity as a risk factor for COVID-19: an overview Critical Reviews in Food Science and Nutrition 2021 61 13 2262 2276 10.1080/10408398.2020.1775546 32539446

[19] Carvalho-Schneider C Laurent E Lemaignen A Beaufils E Bourbao-Tournois C Follow-up of adults with noncritical COVID-19 two months after symptom onset Clinical Microbiology and Infection 2021 27 2 258 263 10.1016/j.cmi.2020.09.052 33031948 PMC7534895

[20] Kamal M Abo Omirah M Hussein A Saeed H Assessment and characterisation of post-COVID-19 manifestations International Journal of Clinical Practice 2021 75 3 e13746 10.1111/ijcp.13746 32991035 PMC7536922

[21] Xiong Q Xu M Li J Liu Y Zhang J Clinical sequelae of COVID-19 survivors in Wuhan, China: a single-centre longitudinal study Clinical Microbiology and Infection 2021 27 1 89 95 10.1016/j.cmi.2020.09.023 32979574 PMC7510771

